# Low-Carbohydrate Nutrition Counseling With Continuous Glucose Monitoring to Improve Metabolic Health Among Veterans With Type 2 Diabetes: Pilot Quality Improvement Initiative Study

**DOI:** 10.2196/75672

**Published:** 2025-12-15

**Authors:** Cassie D Turner, Kishor Patel, Katherine Freeman, Lyndsay Ruff, Jamie Michaels, Timothy Bodnar, Laura R Saslow, James Henderson, Lauren Oshman, Caroline R Richardson, Devvrat Malhotra, A Mark Fendrick, Garth Strohbehn, Dina H Griauzde

**Affiliations:** 1 Department of Internal Medicine, Division of General Medicine, Medical School, University of Michigan, 2800 Plymouth Road, Building 16, Room 16-371C, Ann Arbor, MI, 48109, United States, 1 6038601066; 2 Department of Learning Health Sciences, Medical School, University of Michigan, Ann Arbor, MI, United States; 3 VA Ann Arbor Healthcare System, Ann Arbor, MI, United States; 4 Department of Internal Medicine Division of Metabolism, Endocrinology, and Diabetes, Medical School, University of Michigan, Ann Arbor, MI, United States; 5 Institute for Healthcare Policy and Innovation, University of Michigan, Ann Arbor, MI, United States; 6 School of Nursing, University of Michigan, Ann Arbor, MI, United States; 7 Department of Family Medicine, University of Michigan, Ann Arbor, MI, United States; 8 The Warren Alpert Medical School of Brown University and Care New England, Providence, RI, United States; 9 Department of Health Management and Policy, School of Public Health, University of Michigan, Ann Arbor, MI, United States; 10 Center for Value-Based Insurance Design, School of Public Health, University of Michigan, Ann Arbor, MI, United States; 11 Rogel Cancer Center, University of Michigan, Ann Arbor, MI, United States; 12 Department of Internal Medicine Division of Hematology/Oncology, Medical School, University of Michigan, Ann Arbor, MI, United States

**Keywords:** digital health, behavior change, type 2 diabetes, low carbohydrate dietary counseling, veterans, de-prescription, cardiometabolic health, personalized health

## Abstract

**Background:**

One in 4 Veterans who receive care through the Veterans Health Administration has type 2 diabetes (T2D). Dietary carbohydrate restriction can promote weight loss and improve blood glucose control, but Veterans taking certain medications (eg, insulin) may experience serious complications (eg, hypoglycemia) without adequate support and monitoring.

**Objective:**

This study aims to develop and evaluate the feasibility, acceptability, and clinical effectiveness of a pilot low-carbohydrate (LC) nutrition counseling program guided by continuous glucose monitoring (CGM) for Veterans with T2D receiving insulin (ie, LC-CGM).

**Methods:**

This is a pragmatic, nonrandomized, pre-post quality improvement pilot program. Eligible patients were Veterans with T2D who were prescribed ≥3 daily injections of insulin. The 24-week LC-CGM program consisted of virtual visits with a registered dietitian (RD) and clinical pharmacy practitioner (CPP); CGM data were used to guide tailored nutrition counseling and de-escalation or cessation of glucose-lowering medications. To evaluate changes from baseline, intention-to-treat analyses were conducted for all enrollees, with separate analyses for program completers. Primary outcomes were program feasibility and acceptability (ie, program enrollment and completion rates and mean number of RD and CPP visits). Secondary outcomes included mean weight change, percent weight loss, achievement of ≥5% and ≥10% weight loss, change in glucose-lowering medication use, and change in laboratory measures (eg, hemoglobin A_1c_ [HbA_1c_]).

**Results:**

Program evaluation occurred from March 19, 2021, to May 3, 2024. Among 43 Veterans referred to the LC-CGM program, 38 (88%) enrolled. Most were men (37/38, 97%), white (29/38, 76%), with an average age of 63.7 (SD 9.6) years. Mean BMI and HbA_1c_ were 38.1 (SD 5.8) kg/m^2^ and 7.8% (SD 1.3). Of 38 enrollees, 27 (71%) completed the program. Enrollees averaged 9.5 (SD 3.3) RD visits and 12.8 (SD 4.7) CPP visits. In intention-to-treat analyses, mean weight change was −11.5 kilograms (SD 8.7; 95% CI −14.4 to −8.6), corresponding to 9.5% weight loss (SD 7.2; 95% CI −14.9 to −4.2), with 58% (22/38) achieving ≥5% weight loss and 32% (12/38) achieving ≥10% weight loss. Overall, use of glucose-lowering medications decreased from 3.5 (SD 0.8) per patient at baseline to 2.4 (SD 0.9) per patient at 24 weeks (*P*<.001), with 72% (26/36) of Veterans discontinuing short-acting insulin and 50% (18/36; *P*<.001) discontinuing long-acting insulin. Use of glucagon-like peptide-1 receptor agonists increased from 39% (15/38) at baseline to 61% (23/38) at 24 weeks (*P*=.02). Among program completers (n=27), mean percent weight loss was −11.8% (SD 6.5) and median HbA_1c_ decreased by 0.7% (95% CI −0.9 to −0.3; *P*=.001).

**Conclusions:**

This pilot program provides preliminary evidence that supports feasibility, acceptability, and clinical effectiveness among Veterans with T2D. Additional research is needed to rigorously test longer-term clinical and cost-effectiveness among a larger cohort of eligible Veterans.

## Introduction

Globally, type 2 diabetes (T2D) is a leading cause of morbidity, mortality, and health care spending [1-3]. In the US, rates of T2D are particularly high among Veterans who receive care through the Veterans Health Administration (VHA), with estimates suggesting that 25% of Veterans (1.6 million) have the condition [4], due, in part, to high rates of overweight and obesity (78%) [5]. Among people with T2D, weight loss can improve glycemic control, reduce or eliminate the need for antihyperglycemic medications including insulin, and improve key laboratory measures of cardiometabolic health, including lipids, inflammatory markers, and insulin resistance [6,7].

Certain dietary interventions, including low- and very low-carbohydrate (VLC) ketogenic diets, defined as <130 grams of total carbohydrate per day and <50 grams of total carbohydrate per day, respectively [8], may be particularly effective for supporting weight loss and glycemic control among patients with T2D [9,10]. Accordingly, Veterans Affairs (VA) and Department of Defense Clinical Practice Guideline Recommendations for the management of T2D support patient-centered use of carbohydrate-restricted eating patterns [11], which may be preferred by some Veterans with T2D [12] and prediabetes [13]. However, carbohydrate-restricted eating patterns are not routinely offered to Veterans with T2D due, in part, to limited strategies to support their effective and safe implementation in real-world clinical settings. Specifically, patients commonly require intensive, personalized nutrition counseling to ensure adherence to a well-formulated, low-carbohydrate diet and timely adjustments of certain medications (eg, insulin, sulfonylureas, and antihypertensive agents) to mitigate risks of hypoglycemia and hypotension [14-16].

Novel and scalable strategies are thus needed to enhance patients’ access to effective carbohydrate-restricted nutrition counseling while minimizing potential risks. Continuous glucose monitoring (CGM) may be one highly promising tool to accomplish these dual priorities [17-19]. CGM provides real-time data on blood glucose levels and insight into how specific foods and behaviors (eg, physical activity, sleep, and stress) influence individual patients’ glycemic status. CGM is a key component of effective digital health interventions for T2D self-management [20] and can improve glycemic control while reducing the risk of hypoglycemia [21-24].

The use of CGM to support safe and effective carbohydrate-restricted nutrition counseling for Veterans aligns with existing local VA priorities to deliver patient-centered, evidence-based treatment to Veterans using team-based care models that support innovation and continuous improvement [25]. To advance these priorities, we aimed to develop and evaluate a pilot quality improvement (QI) program to deliver tailored, low-carbohydrate nutrition counseling guided by CGM to Veterans with T2D in a single VA health care system. We hypothesize that this program will be feasible and acceptable among Veterans and that it will support weight loss and improved glycemic control while reducing the use of glucose-lowering medications. These data may inform future efforts to support a weight-focused approach to T2D management within VHA.

## Methods

### Study Design

We conducted a pragmatic, nonrandomized, pre-post evaluation of a QI pilot to examine the feasibility and effectiveness of low-carbohydrate nutrition counseling guided by continuous glucose monitoring (referred to hereafter as LC-CGM) among Veterans with T2D receiving insulin. The intervention was 24 weeks in duration, and all procedures, including patient recruitment, enrollment, education, and monitoring, were integrated into usual clinical care operations. The program evaluation was conducted from March 19, 2021, to May 3, 2024, with data analyzed from February 2024 through March 2025.

### Setting

This program was implemented within the VA Ann Arbor Healthcare System, which includes the Lieutenant Colonel Charles S. Kettles VA Medical Center and 6 other freestanding VA outpatient health clinics serving a diverse patient population throughout southeast Michigan and northwest Ohio [26]. The program was delivered remotely using the VA Video Connect (VVC) platform or telephone, depending on patients’ preferences.

### QI Team and Training

The QI team consisted of 1 clinical pharmacist practitioner (CPP), 2 registered dietitians (RD), 2 primary care physicians, and 1 endocrinologist. The CPP dedicated 0.1 full-time equivalent to 4 hours per week, and the RDs dedicated a combined 0.15 full-time equivalent to 6 hours per week to the program; the physicians did not have a dedicated program effort and devoted less than 1 hour per week, on average, to supporting the CPP and RDs. RDs and CPPs were trained to support Veterans’ adherence to a low-carbohydrate diet using materials previously developed by the study team [13,27], online resources [28], and case-based discussions during monthly team meetings.

### Participants and Recruitment

For this pilot program, we aimed to recruit at least 20 Veterans meeting study inclusion criteria; we limited enrollment to 10 Veterans at one time due to staffing capacity. Inclusion criteria were: (1) Veterans with T2D, (2) use of ≥3 insulin injections per day (based on the VA criteria for use of CGM during the program evaluation period), (3) BMI≥25 kg/m^2^, (4) desire to lose weight or improve metabolic health, (5) willingness and ability to use CGM technology, (6) willingness and ability to modify dietary intake to follow a carbohydrate-restricted eating pattern, (7) willingness to self-weigh at least once weekly and report weight information to the program team, and (8) willingness to engage in regular virtual visits with clinical team members, including a RD and clinical pharmacist. Exclusion criteria were (1) type 1 diabetes, (2) chronic kidney disease stage 4 or greater, (3) advanced heart failure, and (4) pregnant or breastfeeding.

Primary care clinicians or CPPs identified potentially eligible patients during routine clinical encounters, assessed their interest in LC-CGM program participation, and alerted QI team members via an electronic health record (EHR) message if patients desired to learn more about the opportunity. Within 2 weeks of receiving the EHR alert, a QI team member (DHG or KF) called patients by phone to discuss the program, address any questions or concerns, and initiate enrollment if desired; the enrollment phone script is shown in Multimedia Appendix 1.

### Intervention

#### Visits With CPP

The initial visit with the CPP was 30 minutes in duration. During this visit, the clinical pharmacist reviewed (1) patients’ active medications, including insulin dosing regimen, (2) patients’ adherence to prescribed medications, and (3) LC-CGM program expectations (eg, regular attendance at virtual visits) and goals (eg, to reduce dietary carbohydrate intake to support weight loss and glycemic control with fewer medications). The CPP also ensured patients appropriately used CGM, including regularly transmitting data to the clinical review platform; use of CGM tracking features for dietary intake, weight, and physical activity was encouraged but not required.

The CPP informed patients of common side effects that may occur when transitioning from a high-carbohydrate to a low-carbohydrate eating pattern (eg, headache, fatigue, lightheadedness, constipation, and muscle cramps) and strategies to minimize these events (eg, gradual carbohydrate reduction with increased intake of water, electrolytes, and nonstarchy vegetables). Patients were also informed of signs and symptoms of hypoglycemia and hypotension, if taking antihypertensive medication, and counseled on strategies to prevent and treat these events [29]. Veterans were advised to contact the CPP by phone or EHR-based portal messaging if (1) they experienced signs or symptoms of hypoglycemia or hypotension, (2) fasting blood sugar levels <100 mg/dL, or (3) blood pressure <110/70 mm Hg, if using antihypertensive medication. Patients were advised to seek emergency care for severe symptoms.

Use of VLC diets (<50 g of total carbohydrate per day) is relatively contraindicated in patients taking sodium glucose cotransporter-2 (SGLT2 inhibitors) due to the rare risk of euglycemic diabetic ketoacidosis [30]. SGLT2 inhibitors may be indicated for patients with T2D, renal disease, or cardiovascular disease. In general, if LC-CGM patients were prescribed an SGLT2 inhibitor for T2D in the absence of cardiovascular or renal disease and desired to follow a VLC diet, the SGLT2 inhibitor was discontinued during the first visit with the CPP. If patients were prescribed an SGLT2 inhibitor for cardiovascular or renal disease, the SGLT2 inhibitor was generally continued, and patients were counseled to consume 70‐100 grams of total carbohydrate per day. Additional antihyperglycemic changes, including a reduction in insulin dose, were informed by clinical practice guideline recommendations and patients’ baseline glycemic control [31].

Subsequent 30-minute visits with the CPP were scheduled at 3, 6, 12, 18, and 24 weeks. Between scheduled visits, the CPP reviewed patients’ CGM data at least once per week and initiated outreach via telephone call or EHR portal message to adjust medications, if needed. The CPP communicated medication changes and issues of concern to the LC-CGM program RD and, if needed, to QI team physicians, via EHR-based messaging or phone call.

#### Visits With RD

The first RD visit was scheduled within 2 weeks following the initial CPP visit. All patients met virtually with the RD once per week for 4 consecutive weeks; visit frequency was then tailored to individual patients’ needs and preferences. For example, patients struggling to follow a carbohydrate-restricted eating pattern continued with weekly visits, whereas patients who easily transitioned to this eating pattern reduced visit frequency to every other week or once monthly. All visits were 30 minutes in duration. In general, RDs taught patients to follow a VLC diet consisting of 20‐35 net carbohydrates per day (net carbohydrates=total carbohydrates – fiber in g). Patients prescribed an SGLT2 inhibitor were advised to consume 70‐100 grams of total carbohydrate per day.

Consistent with our team’s prior work among Veterans with prediabetes [13], LC-CGM patients were not provided with an explicit calorie limit. Rather, patients were encouraged to eat when hungry and stop when satisfied. Patients were taught to change 1 meal per week from a high- to a VLC option, starting with breakfast and aiming for approximately 10 grams of net carbohydrates per meal or less. Patients were encouraged to consume foods such as meat, fish, poultry, eggs, cheese, seeds, nuts, leafy greens, nonstarchy vegetables, and some fruits (eg, berries). Patients who preferred plant-based sources of protein were encouraged to consume tofu and tempeh. In general, participants were not provided with specific meal plans but rather provided with a variety of low-carbohydrate alternatives to high-carbohydrate foods and meals.

Patients were encouraged to maintain food logs; these were reviewed in conjunction with CGM data during RD visits. RDs used these data to help patients understand how specific foods influenced their blood sugar levels. If patients reported signs or symptoms of hypoglycemia or if CGM data revealed blood glucose <70 mg/dL, the RD reiterated hypoglycemia management strategies and communicated directly by telephone or a secure messaging platform with the CPP or QI team physician to ensure timely adjustments to patients’ anti-hyperglycemic regimen.

#### Discharge From LC-CGM Program

Veterans were able to stop program participation at any time if they did not desire to use CGM or receive carbohydrate-restricted dietary counseling. Veterans were discharged from the program if they were unable to complete regular follow-up visits or engage in self-monitoring with CGM. Veterans were also discharged after completing the 6-month program. In all cases, Veterans’ primary care teams were notified through EHR-based messaging of patients’ progress and clinical needs at the time of LC-CGM discharge (eg, ongoing follow-up with primary care CPP). All patients who completed the LC-CGM program were offered optional participation in a virtual, once-monthly low-carbohydrate maintenance group.

### Data Collection

Prior to initiating the LC-CGM program, patients completed baseline fasting laboratory testing, including a comprehensive metabolic panel, hemoglobin A_1c_ (HbA_1c_), lipids, and c-peptide (to confirm endogenous insulin production). At 24 weeks, patients were asked to complete repeat laboratory testing for HbA_1c_ and fasting lipids.

Patients were advised to self-weigh at least once weekly [24]. Veterans without access to a home scale were provided with one, per usual clinical procedures. Patients reported weight change data to the RD during scheduled visits; these data were entered into patients’ medical records.

The following data were collected from the EHR: sociodemographic factors (age, sex, race, and ethnicity), anthropometric measures (height, weight, and BMI), laboratory test results (HbA_1c_, total cholesterol, high-density lipoprotein [HDL], low-density lipoprotein [LDL], and triglycerides), and T2D medications prescriptions, including biguanides (eg, metformin), sulfonylureas, thiazolidinediones, meglitinides (eg, repaglinide), glucagon-like peptide 1 receptor agonists (GLP-1 RAs), dipeptidyl peptidase-4 (DPP-4) inhibitors, and SGLT2 inhibitors, and insulins. We also collected data on insulin type (eg, short-acting and long-acting) and total daily dosages (TDD) for each type.

### Outcome Measures

Primary outcomes include measures of feasibility and acceptability:

1. Program enrollment rate, defined as the number of referred patients who enrolled in the program divided by the total number of referred patients.
2. Mean program engagement, defined as the number of weeks engaged in LC-CGM.
3. Program completion rate, defined as the number of patients who completed the 24-week program divided by the number of program enrollees.
4. Mean number of CPP visits among all program enrollees and among program completers.
5. Mean number of RD visits among all program enrollees and among program completers.

Secondary outcomes include the following clinical effectiveness measures:

1. Mean change in self-reported weight at 4, 12, and 24 weeks compared to baseline. Veterans were advised to self-weigh at least once weekly and report the most recent measure at each scheduled visit.
2. Mean percent weight change at 4, 12, and 24 weeks compared to baseline.
3. Achievement of ≥5% and ≥10% weight loss at 4, 12, and 24 weeks, defined as the percentage of participants who achieved these weight thresholds divided by the total number of participants.
4. Change in glucose-lowering medication use at 24 weeks.
5. Change in TDD of insulin at 24 weeks.
6. Mean change in HbA_1c_, LDL, HDL, and triglycerides among program completers at 24 weeks.

### Statistical Analysis

We evaluated weight and medication change outcomes for all program enrollees using data available in their final LC-CGM clinic note. Laboratory data were evaluated if available within 30 days of the final LC-CGM visit.

We assessed baseline characteristics of those who enrolled and compared baseline characteristics of those who enrolled and completed the program to those who enrolled but did not complete the program.

We computed descriptive statistics for collected variables, including means, medians, and IQRs for continuous variables and counts and proportions for categorical variables. To assess differences, we conducted intention-to-treat analyses and used the last observation carried forward method for missing data from noncompleters. We also assessed differences among those who completed the program. For continuous variables, we used the Shapiro-Wilk test to assess the normality of the distribution of differences. We compared baseline to 24-week changes using paired samples 2-tailed *t* tests or paired Wilcoxon exact signed rank tests with continuity correction. To compare differences in proportions, we constructed contingency tables and completed McNemar difference in paired proportions tests with continuity correction to improve the approximation of the discrete probability distribution with a continuous one [32]. To determine statistical significance, we used a 2-sided *P*<.05. All analyses were completed in R (version 4.1.3; R Core Team).

### Ethical Considerations

This program was reviewed by the VA Ann Arbor Healthcare System’s Quality Management Office, which reviews QI program applications to determine whether Institutional Review Board oversight is required. This project was approved as a nonresearch, QI initiative. As such, patients were informed that participation was voluntary and were also provided the opportunity to verbally accept or decline participation prior to data collection (Multimedia Appendix 1). To safeguard patient information, extracted data were deidentified and aggregated for analysis.

## Results

### Overview

**Figure 1.**
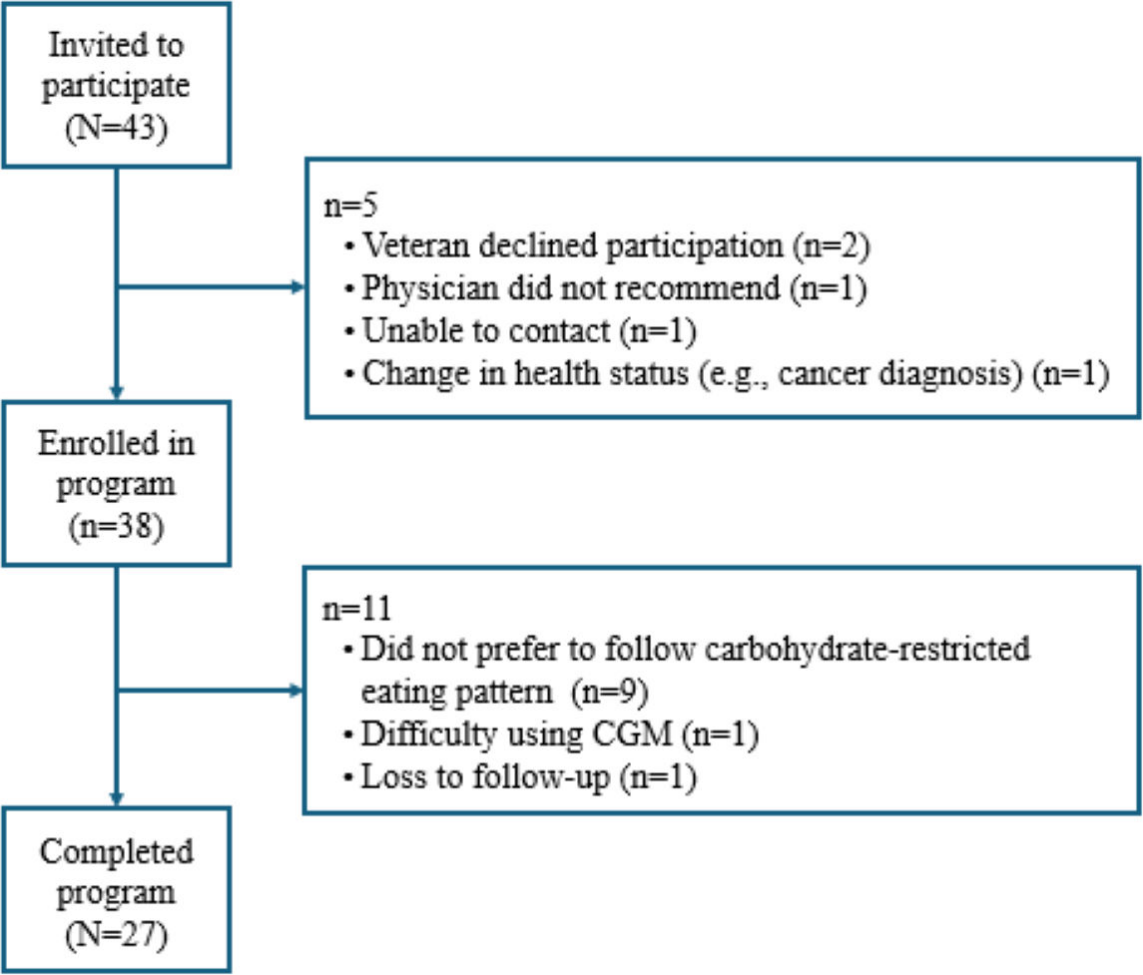
Study flow diagram. CGM: continuous glucose monitoring.

Overall, 43 Veterans were referred to the LC-CGM program during the evaluation period and 38 (88%) enrolled. Among program enrollees, 27/38 (71%) completed the 24-week program. Reasons for program nonenrollment and noncompletion are shown in Figure 1. The main reason for program noncompletion was Veterans’ preference not to follow a carbohydrate-restricted eating pattern (9/11, 81.8%).

### Baseline Characteristics

Baseline sociodemographic and clinical characteristics for program enrollees, stratified by program completion status, are shown in Table 1. Most program enrollees were men (37/38, 97%), white (29/38, 76%), and the average age was 63.7 (SD 9.6) years. Mean BMI was 38.1 (SD 5.8) kg/m^2^ and mean HbA_1c_ was 7.8% (SD 1.3%). There were no significant differences in baseline characteristics of program completers versus noncompleters. Overall, patients were prescribed an average of 3.5 (SD 0.8) glucose-lowering medications, and all were prescribed insulin.

**Table 1.** Baseline characteristics of program enrollees, stratified by program completers and noncompleters.

Variables	Program enrollees (n=38)	Program completers (n=27)	Program noncompleters (n=11)	*P* value
Sociodemographic characteristics				
Age (years)				.91
Mean (SD)	63.7 (9.6)	63.7 (9.4)	63.7 (10.5)	
Median (IQR)	67 (56‐72)	66 (56‐72)	67 (58‐72)	
Sex, n (%)				
Male	37 (97)	26 (96)	11 (100)	>.99
Race, n (%)				.14
Black or African American	3 (8)	1 (4)	2 (18)	
White or Caucasian	29 (76)	22 (81)	7 (64)	
Other	1 (3)	0 (0)	1 (9)	
Missing	5 (13)	4 (15)	1 (9)	
Ethnicity, n (%)				.80
Hispanic	2 (5)	1 (4)	1 (9)	
Non-Hispanic	34 (89)	24 (89)	10 (91)	
Missing	2 (5)	2 (7)	0 (0)	
Anthropometric characteristics				
Weight (kg)				.50
Mean (SD)	121.2 (24)	122.7 (25.1)	117.5 (21.5)	
Median (IQR)	115 (101.4‐137.6)	119.1 (100.8‐142.4)	112.9 (105.5‐128.1)	
BMI (kg/m^2^)				.92
Mean (SD)	38.1 (5.8)	38.2 (6.2)	37.9 (5.1)	
Median (IQR)	37.2 (34.1‐42.5)	36.6 (34‐42.7)	37.7 (34.7‐40.6)	
Cardiometabolic laboratory data				
HbA_1c_ ^a^(%)				.30
Mean (SD)	7.8 (1.3)	7.7 (1.5)	8 (0.9)	
Median (IQR)	7.8 (7.1‐8.3)	7.5 (7‐8.2)	8 (7.5‐8.2)	
HDL^b^ (mg/dL)				.07
Mean (SD)	36.6 (8)	35 (7.2)	40.8 (8.8)	
Median (IQR)	35 (31‐38)	34 (30.5‐37)	37 (33.5‐49.8)	
LDL^c^ (mg/dL)				.40
Mean (SD)	76.1 (40.5)	74.1 (43)	80.7 (35.4)	
Median (IQR)	61.5 (43.2‐94.8)	56 (40‐92.2)	77 (56.5‐94.5)	
Cholesterol (mg/dL)				.64
Mean (SD)	156.4 (54)	157.3 (60.2)	153.8 (34.5)	
Median (IQR)	137 (118‐174)	135 (113.5‐178.5)	143.5 (133‐168.2)	
Triglycerides (mg/dL)				.20
Mean (SD)	232 (135)	250.7 (145.6)	182 (88.8)	
Median (IQR)	199 (149‐281)	201 (160.5‐330)	175 (101.8‐254.5)	
Glucose-lowering medications				
Number of prescriptions				
Mean (SD)	3.5 (0.8)	3.4 (0.8)	3.6 (0.9)	.78
Median (IQR)	3 (3‐4)	3 (3‐4)	3 (3‐4)	.78
Patients with ≤2 prescriptions, n (%)	4 (11)	3 (11)	1 (9)	.80
Patients with 3 prescriptions, n (%)	16 (42)	11 (41)	5 (45)	.80
Patients with 4 or more prescriptions, n (%)	18 (47)	13 (48)	5 (45)	.80
Glucose-lowering medication type, n (%)				
Long-acting insulin	36 (95)	26 (96)	10 (91)	.50
Short-acting insulin	35 (92)	26 (96)	9 (82)	.20
U-500 insulin	2 (5)	1 (3.7)	1 (9)	.50
Biguanide (metformin)	25 (66)	18 (67)	7 (64)	>.99
Sodium-glucose cotransporter 2 inhibitor	17 (45)	12 (44)	5 (45)	>.99
Glucagon-like peptide 1 receptor agonist	15 (39)	9 (33)	6 (55)	.30
Sulfonylureas	2 (5)	1 (4)	1 (9)	.50
Dipeptidyl peptidase 4 inhibitor	0 (0)	0 (0)	0 (0)	—^d^
Meglitinides	1 (3)	1 (4)	0 (0)	—
TDD^e^ of insulin in units, mean (SD)				
Long-acting insulin	78.4 (39.3)	76.5 (39.2)	83.4 (41.2)	.70
Short-acting insulin	42.5 (28.3)	40.9 (27.4)	46.9 (32)	.60
U-500 concentrated insulin	420 (113.1)	340 (—)	500 (—)	>.99

a HbA_1c_: hemoglobin A_1c_.

b HDL: high-density lipoprotein.

c LDL: low-density lipoprotein.

d Not available.

e TDD: total daily dose.

### Primary Outcomes

The number of program enrollees and completers is shown in Figure 1. Among program enrollees, the mean number of weeks engaged in the LC-CGM was 20.7 (SD 5.6). As shown in Table 2, the mean number of program visits with the RD was 10.7 (SD 2.4) among program completers and 6.5 (SD 3.4) among program noncompleters (*P*<.001). The mean number of program visits with the CPP was 15.1 (SD 3) among program completers and 7.2 (SD 2.8) among program noncompleters (*P*<.001).

**Table 2.** Engagement in registered dietitian and clinical pharmacy practitioner visits among program enrollees, stratified by program completers and program noncompleters.

Variables	Program enrollees (n=38)	Program completers (n=27)	Program noncompleters (n=11)	*P* value
Registered dietitian visits				<.001
Mean (SD)	9.5 (3.3)	10.7 (2.4)	6.5 (3.4)	
Median (IQR)	10 (3-17)	10 (7-17)	6 (3-14)	
Clinical pharmacy practitioner visits				<.001
Mean (SD)	12.8 (4.7)	15.1 (3)	7.2 (2.8)	
Median (IQR)	13 (3-24)	15 (11-24)	7 (3-11)	

### Secondary Outcomes

Table 3 shows weight change for the program at 4, 12, and 24 weeks among program enrollees, stratified by program noncompleters who discontinued the program at 4 and 12 weeks.

**Table 3.** Weight change at 4, 12, and 24 weeks among program enrollees, stratified by program noncompleters who discontinued the program at 4 and 12 weeks.

Weight change	Program enrollees	95% CI for difference	Weight change among patients who remained in the program	Weight change among patients who discontinued the program	95% CI for difference	*P* value^a^
Weight change at week 4						
Number of enrollees	38	—^e^	33	5	—	—
Weight change (kg), mean (SD)^b^	−4.5 (4.6)	−6 to −3^c^	−4.9 (4.7)	−0.5 (0.9)	−4.4 (−6.1 to −1.2)	.006
Weight change (kg), median (IQR)^d^	−4.1 (−6.8 to −1.5)	−5.9 to −3.2^c^	−4.5 (−6.9 to −2.7)	−1.3 (1.8)	−3.2 (−6.8 to −0.5)	.03
Weight change (%), mean (SD)	−3.8 (3.7)	—	−4.2 (3.7)	−1.4 (2)	−2.8 (−5.3 to −0.3)	.03
≥5% weight loss, n (%)	13 (34)	—	13 (39)	0 (0)	11‐68	.20
≥10% weight loss, n (%)	1 (3)	—	1 (3)	0 (0)	0‐11.9	>.99
Weight change at week 12						
Number of enrollees	38	—	27	6	—	—
Weight change (kg), mean (SD)^b^	−8.5 (6.2)	−10.5 to −6.4^c^	−9.9 (5.6)	−8.3 (7.8)	−1.6 (−7.1 to 4)	.66
Weight change (kg), median (IQR)^d^	−6.5 (−11.8 to −3.6)	−10.7 to −6.6^c^	−9.5 (−13.5 to −6.3)	−4.3 (−12.1 to −3)	−5.2 (−8.8 to 6.3)	.30
Weight change (%), mean (SD)	−7.2 (5.4)	—	−8.4 (5)	−6.8 (6.3)	−2 (−8 to 5)	.59
≥5% weight loss, n (%)	22 (58)	—	20 (74)	2 (33)	0‐92	.20
≥10% weight loss, n (%)	12 (32)	—	10 (37)	2 (33)	0‐49	>.99
Weight change at week 24						
Number of enrollees	38	—	27	11	—	—
Weight change (kg), mean (SD)^b^	−11.5 (8.7)	−14.4 to −8.6^c^	−14.3 (7.9)	−4.7 (6.9)	−9.6 (−14.9 to −4.2)	.001
Weight change (kg), median (IQR)^d^	−10.9 (−18 to −3.8)	−14.5 to −8.6^c^	−14.7 (−18.9 to −9.3)	−2.7 (−4.3,−0.5)	−12 (−15.7 to −5.4)	.001
Weight change (%), mean (SD)	−9.5 (7.2)	—	−11.8 (6.5)	−3.9 (5.6)	−7.9 (−12.4 to −3.3)	.001
≥5% weight loss n (%)	26 (68)	—	24 (89)	2 (18)	38.6‐100	<.001
≥10% weight loss n (%)	19 (50)	—	17 (63)	2 (18)	9.2‐80.4	.03

a 2-sample *t* test.

b Paired samples *t* test.

c Statistically significant at *P*≤.001.

d Paired Wilcoxon exact signed rank test with continuity correction.

e Not available.

**Figure 2.**
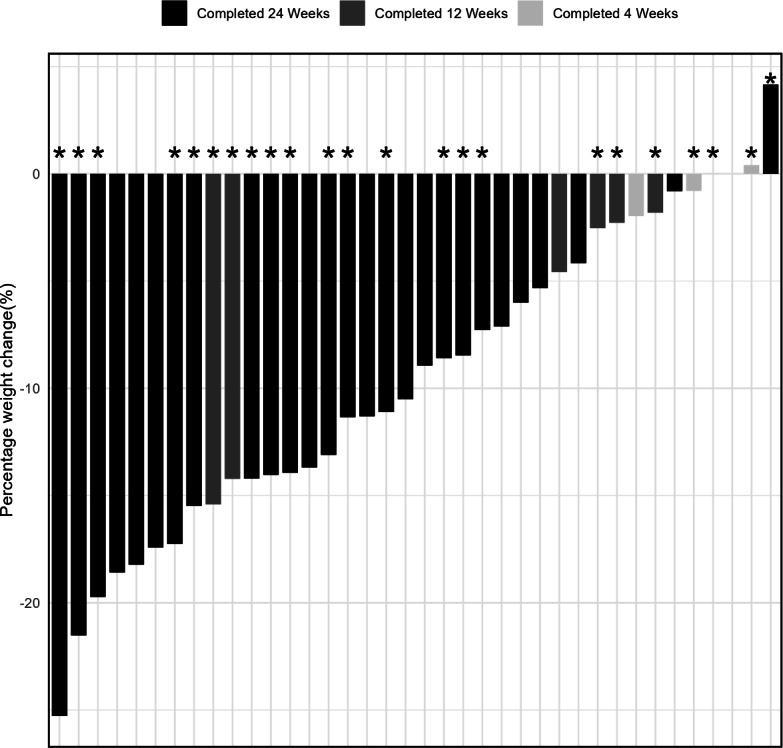
Waterfall plot showing percent weight change for individual program enrollees, with glucagon-like 1 peptide receptor agonist use at 24 weeks designated with an asterisk (*). Each bar corresponds to 1 participant.

Overall, among 38 enrollees, mean weight change was −11.5 kg (SD 8.7) and mean percent weight change was 9.5% (SD 7.2%), with 68% (26/38) achieving ≥ 5% weight loss and 50% (19/38) achieving ≥ 10% weight loss at 24 weeks. Among 27 program completers, mean percent weight loss was 11.8% (SD 6.5%) at 24 weeks, with most achieving ≥5% weight loss (89%, 24/27) and approximately two-thirds achieving ≥10% weight loss (63%, 17/27). At week 4, weight change was significantly greater among those who completed the program compared to those who did not (−4.2% vs 1.4%; *P*=.03). A waterfall plot of weight change for each patient is shown in Figure 2.

Changes in cardiometabolic laboratory data at 24 weeks compared to baseline for program completers who had follow-up laboratory data are shown in Multimedia Appendix 2. Mean HbA_1c_ decreased by 0.7% (CI −0.9% to −0.3%), from 7.7% (SD 1.5%) to 7% (SD 0.9%), with 6 patients (22%) having an HbA_1c_ less than 6.5. On average, patients had significant reductions in total cholesterol (−18.9 mg/dL, SD 28) and triglyceride levels (−80.8 mg/dL, SD 103.2). Mean changes in LDL and HDL cholesterol levels were nonsignificant. In total, 42% (8/19) had a mean increase in LDL cholesterol of 15.8 mg/dL, and 58% (11/19) had a mean decrease in LDL cholesterol of −19.6 mg/dl. Waterfall plots of change in HbA_1c_ and LDL cholesterol for each patient are shown in MultimediaAppendices 3  4.

Table 4 shows changes in glucose-lowering medication use among program enrollees and program completers. At baseline, patients were prescribed an average of 3.5 (SD 0.8) glucose-lowering medications, which decreased to 2.4 (SD 0.9) at 24 weeks. Program completers were prescribed an average of 2.2 (SD 0.9) glucose-lowering medications at 24 weeks. Those who discontinued at 12 weeks were prescribed an average of 2.7 (SD 0.5) glucose-lowering medication, while those who discontinued the program after 4 weeks did not have any medication changes. Among those who completed the program, 21/27 (78%) were prescribed 2 or fewer medications at 24 weeks compared to 3 of 27 (11%) at baseline (Table 4 and Multimedia Appendix 5). Most patients discontinued use of short-acting insulin (24/27, 89%) and long-acting insulin (18/27, 67%) during the program period (Table 4). Changes in TDD of insulin are in Multimedia Appendix 6. Also, fewer patients used SGLT2 inhibitors, sulfonylureas, and meglitinides compared to baseline; however, these changes were nonsignificant (Table 4).

**Table 4.** Change in glucose-lowering medication use among program enrollees and program completers.

	Baseline ITT^a^, n (%), (n=38)	24 Weeks ITT, n (%), (n=38)	Difference (95% CI)^b^	*P* value^b^	Baseline completers n (%), (n=27)	24 Weeks completers n (%), (n=27)	Difference (95% CI)^b^	*P* value^b^
Number of prescriptions, for glucose-lowering medications, mean (SD)	3.5 (0.8)	2.4 (0.9)	−1.1 (−1.4 to −0.73)	<.001	3.4 (0.8)	2.2 (0.9)	−1.5^c^ (−1 to −2)^d^	<.001
Patients with ≤2 prescriptions for glucose-lowering medications, n (%)	4 (11)	23 (61)	50 (31‐69)	<.001	3 (11)	21 (78)	67 (45‐88)	<.001
Biguanides (eg, metformin)	25 (66)	25 (66)	0 (−13 to 13)	.62	18 (67)	18 (67)	0 (−18 to 18)	.62
Short-acting insulin	36 (95)	10 (26)	−66 (−87 to −45)	<.001	26 (96)	3 (11)	−85 (−100 to −68)	<.001
Long-acting insulin	36 (95)	18 (47)	−47 (−67 to −27)	<.001	26 (96)	9 (33)	−63 (−88 to −38)	<.001
Both short- and long-acting insulin	36 (95)	9 (24)	−70 (−90 to −51)	<.001	26 (96)	3 (11)	−88 (−100 to −72)	<.001
U500^d^	2 (5)	0 (0)	−5.3 (−15 to 4.5)	.48	1 (4)	0 (0)	−3.7 (−14.5 to 7.1)	>.99
Sulfonylureas	2 (5)	0 (0)	−5.3 (−15 to 4.5)	.48	1 (4)	0(0)	−3.7 (−14.5 to 7.1)	>.99
DPP-4^e^ inhibitor	0 (0)	7 (18)	-18 (3.5‐33)	.02	0 (0)	6 (22.2)	22.2 (2.8‐41.6)	.04
GLP-1 RA^f^	15 (39)	23 (61)	24 (7.5‐40)	.008	8 (30)	15 (56)	26 (5.8‐46)	.02
SGLT2^g^ Inhibitors	17 (45)	8 (21)	−24 (−43 to −4)	.03	12 (44)	7 (25.9)	−18.5 (−42.9 to 6)	.18
Meglitinides	1 (3)	0 (0)	−2.6 (−10 to 5.1)	>.99	1 (4)	0 (0)	−3.7 (−14.5 to 7.1)	>.99

a ITT: intention to treat.

b McNemar difference in paired proportions test with continuity correction to improve the approximation of the discrete probability distribution with a continuous one.

c Paired Wilcoxon exact signed rank test with continuity correction due to *P* value <.05 on Shapiro-Wilk test (suggesting distribution of differences is not normal). Median difference and confidence interval for median are reported instead of the mean difference for total number of anti-hyperglycemic medications (mean of −1.3 and SD of 1.1) and short-acting insulin (mean of −36.1 mg/dl and SD of 28.8).

d U500 is highly concentrated insulin, 500 units/mL, typically used for highly insulin-resistant patients.

e DPP-4: dipeptidyl peptidase 4.

f GLP-1 RA: glucagon-like peptide 1 receptor agonists.

g SGLT2: sodium glucose cotransporter-2.

Notably, of the 27 participants who completed the program, 15 (56%) were prescribed a GLP-1 RA. Those who were prescribed a GLP-1 RA lost an average of 13% body weight (16.4 kg), compared to 10% (11.6 kg) for those (n=12) who were not prescribed a GLP-1 RA. Of the 24 participants who achieved at least 5% body weight loss, 14 (58%) were prescribed a GLP-1 RA at week 2. Of the 17 people who achieved at least 10% body weight loss, 11 (65%) were prescribed a GLP-1 RA at week 24. Figure 2 shows patient-level weight change stratified by prescription of GLP-1 RA for those who completed the program.

### Adverse Events and Side Effects

There were no serious adverse events during the evaluation period. Overall, 18% of patients (7/38) experienced mild (level 1) hypoglycemia (blood glucose level between 54 mg/dL and 70 mg/dL), which was managed as an outpatient using standard protocols and medication de-escalation [29]. Side effects included mild gastrointestinal upset, including belching (n=1), diarrhea with use of semaglutide, resulting in medication discontinuation (n=1), nausea due to metformin, resulting in dose de-escalation (n=1), and increased feelings of anger (n=1).

## Discussion

### Principal Results and Comparison With Prior Work

This QI pilot evaluated the feasibility, acceptability, and clinical effectiveness of tailored low-carbohydrate nutrition counseling supported by continuous glucose monitoring (LC-CGM) among Veterans with T2D using insulin. Most Veterans referred to the LC-CGM program enrolled (38/43, 88%) and, among enrollees, mean engagement was 20 weeks, with approximately 3-quarters (27/38, 71%) completing the 24-week program. Additionally, the LC-CGM program appeared to be an effective and safe intervention to support significant reductions in weight, HbA_1c_, and use of glucose-lowering medications, including insulin.

Our team previously observed high levels of engagement (77%) in a local pilot evaluation of a group-based, VLC diabetes prevention program for Veterans with prediabetes [13]. In contrast, only 25% of Veterans who enroll in the VHA’s MOVE! Weight Management Program, a comprehensive lifestyle intervention that teaches Veterans to follow a conventional, calorie-restricted diet, complete 8 or more sessions within 6 months [33]. Taken together, these findings support prior literature suggesting that some Veterans may prefer a carbohydrate-restricted approach to dietary change [12,13,34].

Prior work has similarly demonstrated the effectiveness of VLC nutrition counseling among individuals who chose to participate in a continuous care intervention (Virta Health) consisting of individualized low-carbohydrate nutrition counseling focused on achieving nutritional ketosis, health coaching, and remote data monitoring (eg, weight and finger-stick blood glucose) [35,36]. A long-term (24-month) evaluation of this intervention demonstrated high levels of program retention (194/262, 74%), with sustained reductions in weight (−10%), HbA_1c_ (−0.9%), and prescriptions for T2D medications (−67%) compared to baseline (vs no change in the usual care group) [37]. In 2019, the Department of Veterans Affairs initiated a partnership with Virta Health to pilot test the intervention among Veterans with T2D and overweight or obesity to provide “a more comprehensive approach to care.” [38] At 24 months, weight loss was significantly greater among Veterans in the intervention versus waitlist control group (−12 kg vs 3 kg). Both groups had a reduction in HbA_1c_ (−0.79 % and −0.59% respectively). However, among intervention participants, this was achieved through dietary carbohydrate restriction with concomitant de-escalation of glucose-lowering medications; control participants required the use of more glucose-lowering medications to reduce HbA_1c_ [39]. Accordingly, monthly per-patient outpatient and pharmacy drug costs were significantly lower among intervention versus usual care participants [39]. Additional work is needed to evaluate the cost-effectiveness of low-carbohydrate nutrition counseling programs, including LC-CGM, which often require intensive support in the near term but may ultimately reduce health care and medication use.

Although overall use of glucose-lowering medications, including insulin, decreased during the study period, there was a significant increase in use of GLP-1 RAs, with 39% (15/38) of LC-CGM patients using a GLP-1 RA at baseline and 61% (23/38) using a GLP-1 RA at 24 weeks. This finding contrasts with recent literature demonstrating that carbohydrate-restricted diets can be used to de-escalate or avoid GLP-1 RA prescriptions [40] and support T2D remission, defined as HbA_1c_ <6.5% at least 3 months after stopping glucose-lowering medications [9,41]. This is due, at least in part, to 2 key factors. First, the LC-CGM program was intended to optimize Veterans’ glucose-lowering medication regimen to support weight loss and improved metabolic health; T2D remission was not an explicit goal of this program, though 22% (6/27) of program completers achieved HbA_1c_ <6.5% (Multimedia Appendix 7 for medication use among participants with HbA_1c_ <6.5% at 24 wk). Second, this was a highly pragmatic quality initiative with inclusion of patients who are less likely to achieve T2D remission (eg, older age, suboptimal glycemic control, use of multiple glucose-lowering medications, and the use of insulin) [42]. Notably, mean weight loss among Veterans prescribed a GLP-1 RA in the LC-CGM program (–10.2%) was greater than expected from clinical efficacy trials of GLP-1 RAs among patients with T2D, with the greatest weight loss (−9.6%) achieved with semaglutide [43]. Given the high cost of GLP-1 RAs [44] and prior reports of their attenuated effectiveness in general practice settings [45,46], further work is warranted to examine the clinical and cost-effectiveness of the LC-CGM program in a large-scale trial.

A key strength of the LC-CGM program is its use of existing VA infrastructure and resources, including clinical team members, and its alignment with VHA’s priorities to deliver team-based, coordinated, and patient-centered care to help Veterans achieve their personal health goals [47,48]. Due to the intensive nature of the program, inclusion of Veterans at high risk for adverse events (eg, hypoglycemia and hypotension), and need for proactive, early deprescribing of glucose-lowering medications [16,49], we were able to support a maximum of 10 Veterans at a time with 6 hours per week of dedicated RD effort and 4 hours per week of dedicated CPP effort. Additional work is needed to explore opportunities to increase LC-CGM capacity; this may include use of group-based and asynchronous education sessions, integration of digital health interventions (eg, text messages and artificial intelligence) [50], and inclusion of patients at lower risk for hypoglycemia (eg, individuals with prediabetes, new diagnosis of T2D, or T2D not requiring insulin) [51]. If offered as a group-based program, LC-CGM’s visit frequency and overall duration would align with other group-based comprehensive lifestyle interventions, including the Centers for Disease Control and Prevention’s Diabetes Prevention Program and the VHA’s MOVE! Weight Management Program. Notably, MOVE! is also offered as a one-on-one program, with 12 sessions more than 12 months with a dietitian. LC-CGM’s involvement of a CPP increases staffing requirements compared to MOVE! but it may not be required if the LC-CGM program was offered to patients at lower risk for hypoglycemia. Additional work is needed to understand if LC-CGM’s short-term, resource-intensive nature is offset by future reductions in health care use, including primary care visits, emergency department encounters, and inpatient hospitalizations.

### Limitations

This study had several limitations. First, this was a QI program evaluation from a single VA health care system; the results may not be generalizable to other VA sites or non-Veteran populations. Second, due to the pragmatic nature of the LC-CGM program referral process, we do not know the number of Veterans who were offered LC-CGM participation but declined referral; this information should be collected in a larger scale trial to further discern the program’s acceptability. Third, we did not evaluate outcomes beyond 6 months, and we are therefore unable to assess the program’s long-term clinical effectiveness. Prior literature demonstrates sustained clinical benefits of dietary carbohydrate restriction for up to 5 years among a non-Veteran population [52]. Fourth, we did not formally assess dietary carbohydrate intake using validated measures. Rather, we considered reductions in weight, HbA_1c_, and medication use as surrogate indicators of dietary adherence. Fifth, due to the virtual nature of the program, most weight data were obtained through the Veteran’s self-report and may differ from in-clinic measured weight. Notably, prior literature suggests concordance between self-reported and measured weight [53], and objective favorable changes in HbA_1c_ and medication use among LC-CGM patients suggest accuracy of self-reported weight in this cohort. Sixth, we were unable to assess the program’s cost-effectiveness due to the limited, short-term nature of the available data and the nonpublic availability of the VA’s negotiated drug prices, which are often substantially lower than those in non-VA settings. Notably, other intensive lifestyle interventions focused on carbohydrate restriction and medication de-prescription have demonstrated cost savings [54].

### Conclusions

This QI program evaluation demonstrates that individually tailored, LC-CGM can reduce body weight, HbA_1c_, and use of glucose-lowering medications among Veterans with T2D on insulin. This approach aligns with clinical practice guideline recommendations that encourage preference-sensitive nutrition counseling using evidence-based approaches, including low- and VLC diets [11,55]. To inform approaches to T2D management in other VA Medical Centers nationwide and enhance Veterans’ access to guideline-adherent carbohydrate-restricted nutrition counseling, the LC-CGM program should be evaluated in a larger scale implementation initiative with examination of longer term clinical outcomes and program costs, including use of GLP-1 RAs.

## Supplementary material

10.2196/75672 Multimedia Appendix 1 Veterans Affairs low-carbohydrate nutrition counseling guided by continuous glucose monitoring quality improvement pilot recruitment phone script.

10.2196/75672 Multimedia Appendix 2 Cardiometabolic laboratory changes among program completers.

10.2196/75672 Multimedia Appendix 3 Waterfall plot of low-density lipoprotein cholesterol change from baseline to 24 weeks (n=19).

10.2196/75672 Multimedia Appendix 4 Hemoglobin A1C change for participants who completed the program. A total of 27 participants completed the program, and 25 had hemoglobin A1C lab values available.

10.2196/75672 Multimedia Appendix 5 Total number of prescriptions for program completers, baseline to 24 weeks.

10.2196/75672 Multimedia Appendix 6 Change in total daily dose of insulin among program enrollees and program completers.

10.2196/75672 Multimedia Appendix 7 Medications for participants with hemoglobin of <6.5% at 24-week follow-up (n=6).

## Abbreviations

CGM: continuous glucose monitoring
CPP: clinical pharmacy practitioner
DPP-4: dipeptidyl peptidase-4
EHR: electronic health record
GLP-1 RA: glucagon-like peptide-1 receptor agonists
HbA_1c_: hemoglobin A_1c_
HDL: High-density lipoprotein
LC: low carbohydrate
LC-CGM: low carbohydrate nutrition counseling guided by continuous glucose monitoring
LDL: low-density lipoprotein
QI: quality improvement
RD: registered dietitian
SGLT2: sodium glucose cotransporter-2
T2D: type 2 diabetes mellitus
TDD: total daily dosage
VA: Veteran Affairs
VHA: Veterans Health Administration
VLC: very low-carbohydrate
VVC: VA Video Connect platform
