# Supplementary material for: Low-Carbohydrate Nutrition Counseling With Continuous Glucose Monitoring to Improve Metabolic Health Among Veterans With Type 2 Diabetes: Pilot Quality Improvement Initiative Study
Source: JMIR Diabetes. 2025 Dec 15;10:e75672. doi: 10.2196/75672 (PMC12705128; doi:10.2196/75672)
Supplement: Multimedia Appendix 1 [file diabetes-v10-e75672-s001.docx]

**VA LC-CGM quality improvement pilot recruitment phone script**

P*atients who have been told about the program by either a clinic pharmacist or their PCP are called by the QI pilot MD to describe the program goals and expectations*

1. Introduce self
2. Ask to speak with Mr./Mrs. [surname]
3. Explain reason for call once Veteran is on the line. [May schedule call back if Veteran is not available]
   1. I am calling to invite you to participate in a new program for Veterans with type 2 diabetes who use insulin.
   2. The goal of this program is to help patients control their blood sugar, lose weight, and decrease or stop insulin.
   3. To do this, patients will be asked to follow a low-carbohydrate diet and to use a continuous glucose monitor.
4. Ask Veteran if he/she would you like you hear more about the program
   1. If NO: Thank you for your time. Have a great day [End call; document that patient is not interested]
   2. If YES: describe program (#5)
5. Describe program
   1. In this program, patients work closely with a dietitian and pharmacist.
   2. **The dietitian teaches individuals to follow a low carbohydrate diet.**
      1. Carbohydrates are found in foods like bread, pasta, rice, baked goods, breakfast cereals, and fruit. When we eat high-carbohydrate foods, our blood sugar levels increase. Patients with type 2 diabetes often need medications like insulin to help their bodies control their blood sugar levels after eating a high-carbohydrate meal.
      2. Another way to control blood sugar levels is to eat less carbohydrates. This type of eating pattern can be called “low-carb” or “keto.”
      3. Low-carb foods include meat, poultry, fish, eggs, cheese, nuts, seeds, leafy greens, broccoli, cauliflower, peppers, etc. There are low-carb substitutes for most foods (including bread) and there are many excellent low-carb recipes.
      4. If you choose to join this program, a dietitian will work closely with you to gradually transition from a high carb to a low carb meal.
   3. **Do you have any questions about the information that I have shared with you so far?**
      1. **[Answer any questions]**
   4. **The pharmacist works with patients to adjust their diabetes medications.**
      1. Patients who eat fewer carbohydrates are often able to lose weight and control their health conditions with require fewer medications.
      2. A pharmacist advises patients how to adjust their medications.
      3. In addition to following a low-carb diet, patients are asked to wear a continuous glucose monitor to help guide changes to their medications.
   5. **Do you have any questions about the information that I have shared with you so far?**
      1. **[Answer any questions]**
   6. ***We know that for many patients, having diabetes and taking insulin multiple times per day is very challenging. We are motivated to help Veterans in this program to lose weight, improve blood sugar levels, and achieve better health with fewer medications.***
      1. *Does this program sound like something you might be interested to participate in?*
