# Supplementary material for: Low-Carbohydrate Nutrition Counseling With Continuous Glucose Monitoring to Improve Metabolic Health Among Veterans With Type 2 Diabetes: Pilot Quality Improvement Initiative Study
Source: JMIR Diabetes. 2025 Dec 15;10:e75672. doi: 10.2196/75672 (PMC12705128; doi:10.2196/75672)
Supplement: Multimedia Appendix 2 [file diabetes-v10-e75672-s002.docx]

**Table S1.** Cardiometabolic laboratory changes among program completers.

|  | Baseline | | 24 weeks | | Mean Difference (SD)^a^ | 95% CI for Difference^a^ | *P*-value^a^ |
| --- | --- | --- | --- | --- | --- | --- | --- |
|  | N | Mean (SD) | N | Mean (SD) |  |  |  |
|  |  |  |  |  |  |  |  |
| HbA1c^b^ (%) | 27 | 7.7 (1.5) | 25 | 7.0 (0.90) | -0.7 ^b^ | -0.9 to -0.3^b^ | 0.001 |
| Total cholesterol (mg/dl) | 27 | 157.3 (60.2) | 21 | 140.2 (44.3) | -18.9(28.0) | -31.7 to -6.2 | 0.006 |
| Triglycerides (mg/dl) | 27 | 250.7 (145.6) | 21 | 168.1 (87.2) | -80.8 (103.2) | -127.8 to -33.9 | 0.002 |
| HDL (mg/dl) | 27 | 35.04 (7.2) | 21 | 36.5 (7.6) | 1.24 (7.2) | -2.0 to 4.5 | 0.44 |
| LDL (mg/dl) | 24 | 74.12 (43.0) | 21 | 72.0 (41.5) | -4.7 (21.9) | -15.3 to 5.8 | 0.36 |

Abbreviations: sd = Standard Deviation, CI = Confidence Interval

^a^ Paired samples t-test .

^b^ Paired Wilcoxon exact signed rank test with continuity correction due to p-value < 0.05 on Shapiro Wilk test (suggesting distribution of differences is not normal). Median difference and confidence interval for median are calculated. For HbA1c, mean change was -0.8 (SD 1.2)
