# Supplementary material for: Low-Carbohydrate Nutrition Counseling With Continuous Glucose Monitoring to Improve Metabolic Health Among Veterans With Type 2 Diabetes: Pilot Quality Improvement Initiative Study
Source: JMIR Diabetes. 2025 Dec 15;10:e75672. doi: 10.2196/75672 (PMC12705128; doi:10.2196/75672)

**Waterfall plot of LDL Cholesterol change from baseline to 24 weeks (n=19)**

This figure shows the change in LDL among those with follow up labs who completed the program. 19 of the 27 program participants had a 24 week follow up lab value for LDL in the electronic health record.


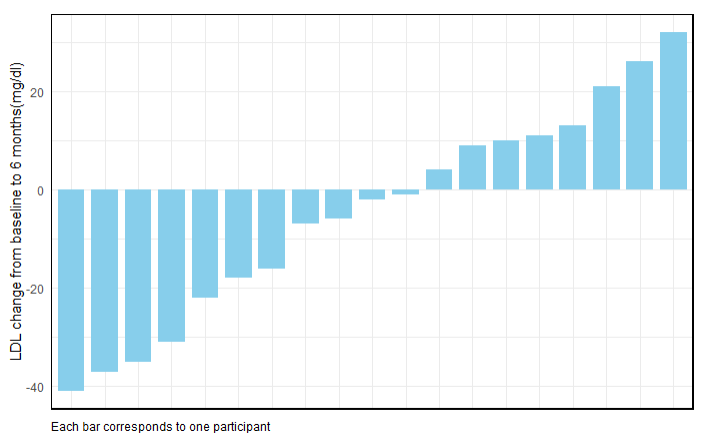

Supplement: Multimedia Appendix 3 [file diabetes-v10-e75672-s003.docx]
