# Supplementary material for: Low-Carbohydrate Nutrition Counseling With Continuous Glucose Monitoring to Improve Metabolic Health Among Veterans With Type 2 Diabetes: Pilot Quality Improvement Initiative Study
Source: JMIR Diabetes. 2025 Dec 15;10:e75672. doi: 10.2196/75672 (PMC12705128; doi:10.2196/75672)

**HbA1c change for participants who completed the program. 27 participants completed the program and 25 had HbA1c lab values available**

6 patients (22%) had an HbA1c less than 6.5 at 24 weeks.


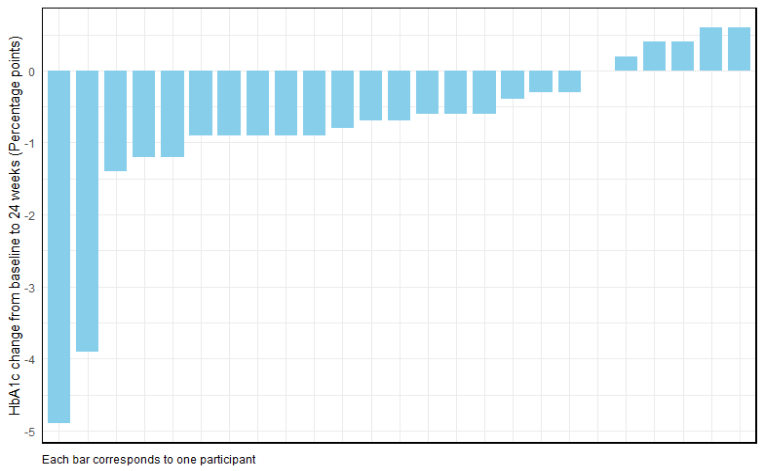

Supplement: Multimedia Appendix 4 [file diabetes-v10-e75672-s004.docx]
