# Supplementary material for: Low-Carbohydrate Nutrition Counseling With Continuous Glucose Monitoring to Improve Metabolic Health Among Veterans With Type 2 Diabetes: Pilot Quality Improvement Initiative Study
Source: JMIR Diabetes. 2025 Dec 15;10:e75672. doi: 10.2196/75672 (PMC12705128; doi:10.2196/75672)
Supplement: Multimedia Appendix 5 [file diabetes-v10-e75672-s005.docx]

**Total number of prescriptions for program completers**, **baseline to 24 weeks**

Change in total medication prescriptions: At baseline patients were taking an average of 3.4 anti-hyperglycemic medications baseline, which decreased to 2.1at follow up.

**
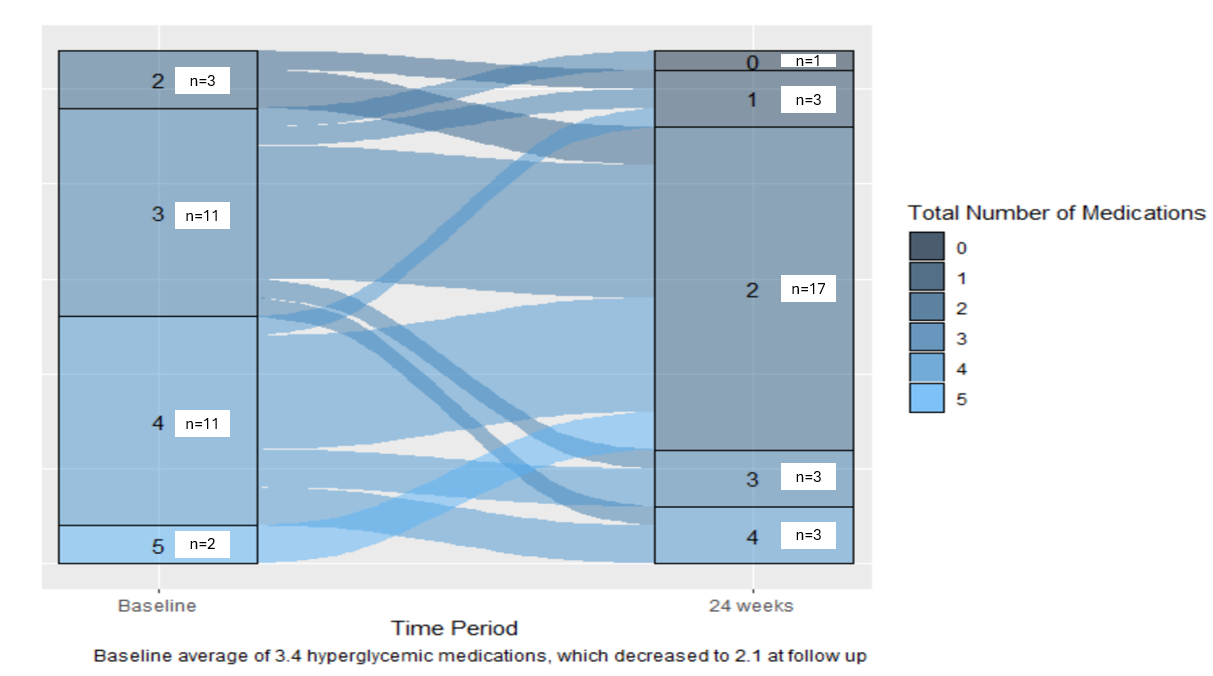
**
