# Supplementary material for: Low-Carbohydrate Nutrition Counseling With Continuous Glucose Monitoring to Improve Metabolic Health Among Veterans With Type 2 Diabetes: Pilot Quality Improvement Initiative Study
Source: JMIR Diabetes. 2025 Dec 15;10:e75672. doi: 10.2196/75672 (PMC12705128; doi:10.2196/75672)
Supplement: Multimedia Appendix 6 [file diabetes-v10-e75672-s006.docx]

Change in total daily dose of insulin among program enrollees and program completers

Among patients who remained on short- and long-acting insulin at 24-week follow-up, the total daily dose was decreased by 92% and 83%, respectively. At 24 weeks, more patients used GLP-1 RAs (26% increase, p = 0.02) and DPP-4 inhibitors (22.2% increase, p = 0.04) compared to baseline.

**Change in total daily dose of insulin among program enrollees and program completers.**

|  |  | Baseline | | 24 Weeks | | Mean Difference (sd) | 95% CI for Difference^a^ | p-value^a^ |
| --- | --- | --- | --- | --- | --- | --- | --- | --- |
|  |  | N | Mean (sd) | N | Mean (sd) |  |  |  |
|  |  |  |  |  |  |  |  |  |
| **Program Enrollees**  **(ITT Analysis)** | |  |  |  |  |  |  |  |
|  | Short-acting insulin tdd (mg/dl) ^b^ | 36 | 42.5 (28.3) | 34 ^b^ | 16.2 (66.73) | -36.8 (30.1) | -47.3 to -26.3. | <0.001 |
|  | Long-acting insulin (mg/dl) | 36 | 78.4 (39.3) | 36 | 24.0 (31.1) | -55.6 (39.0) | -68.7 to -42.4 | <0.001 |
|  | U500  (mg/dl) | 2 | 420 (113.1) | 0 | NA | NA | NA | NA |
| **Program Completers** | |  |  |  |  |  |  |  |
|  | Short-acting insulin tdd (mg/dl) ^b^ | 26 | 39.35(28.04) | 3 | 3.23 (11.41) | -32.0^c^ | -45.0 to -24.0^c^ | <0.001 |
|  | Long-acting insulin (mg/dl) | 26 | 76.5(39.25) | 9 | 13.1 (24.86) | - 63.4 (38.38) | -78.9 to -47.9 | <0.001 |
|  | U500  (mg/dl) | 1 | 340 | 0 | NA | NA | NA | NA |

^a^ Paired samples t-test.

^b^ Missing info due to sliding scale dosages

^c^ Paired Wilcoxon exact signed rank test with continuity correction due to p-value < 0.05 on Shapiro Wilk test (suggesting distribution of differences is not normal). Median difference and confidence interval for median are reported instead of the mean difference for total number of anti-hyperglycemic medications (mean of -1.3 and SD of 1.1) and short-acting insulin (mean of -36.1 mg/dl and SD of 28.8).
