# Supplementary material for: Low-Carbohydrate Nutrition Counseling With Continuous Glucose Monitoring to Improve Metabolic Health Among Veterans With Type 2 Diabetes: Pilot Quality Improvement Initiative Study
Source: JMIR Diabetes. 2025 Dec 15;10:e75672. doi: 10.2196/75672 (PMC12705128; doi:10.2196/75672)
Supplement: Multimedia Appendix 7 [file diabetes-v10-e75672-s007.docx]

|  | SA Insulin | LA Insulin | GLP-1 RA | SGLT2 | Met-formin | 24 Weeks Total Number of Meds | Difference in Number of Meds from Baseline to 24 weeks | Discontinued Meds |
| --- | --- | --- | --- | --- | --- | --- | --- | --- |
| Participant 1 | X | X |  |  |  | 2 | 0 | NA |
| Participant 2 |  |  |  |  |  | 0 | -3 | Meglitinide, SA Insulin, LA Insulin |
| Participant 3 |  |  | X |  | X | 2 | -2 | SA, LA Insulin |
| Participant 4 |  |  | X | X |  | 2 | -3 | Metformin, SA Insulin, LA Insulin |
| Participant 5 |  |  | X |  | X | 2 | -3 | SGLT2, SA Insulin, LA Insulin |
| Participant 6 | X | X |  | X | X | 4 | 0 | NA |
| Total n(%) | 2 (33%) | 2 (33%) | 3 (50%) | 2 (33%) | 3 (50%) |  |  |  |
| *None of the patients with HbA1c < 6.5 were prescribed U500 Insulin, DPP4s, Sulfonylureas, or Meglitinides at 24 weeks | | | | | | | | |

**Table S1.** Medications for participants with HbA1c < 6.5 at 24 week follow up (n=6)
